# Press-fit fixation using autologous bone in the tibial canal causes less enlargement of bone tunnel diameter in ACL reconstruction - a CT scan analysis three months postoperatively

**DOI:** 10.1186/s12891-015-0656-5

**Published:** 2015-08-19

**Authors:** Ralph Akoto, Jonas Müller-Hübenthal, Maurice Balke, Malte Albers, Bertil Bouillon, Philip Helm, Marc Banerjee, Jürgen Höher

**Affiliations:** Department of Trauma and Reconstructive Surgery, Asklepios Clinic St. Georg, Hamburg, Germany; Clinic for diagnostic Radiology and Nuclear medicine, Cologne-Triangle, Cologne, Germany; Department of Trauma and Orthopedic Surgery, University of Witten/Herdecke, Cologne Merheim Medical Center, Cologne, Germany; Clinic for Sports Traumatology at Cologne Merheim Medical Center, Cologne, Germany

## Abstract

**Background:**

Bone tunnel enlargement is a phenomenon present in all anterior cruciate ligament (ACL)- reconstruction techniques. It was hypothesized that press-fit fixation using a free autograft bone plug reduces the overall tunnel size in the tibial tunnel.

**Methods:**

In a prospective cohort study twelve patients who underwent primary ACL reconstruction using an autologous quadriceps tendon graft and adding a free bone block for press-fit fixation (PF) in the tibial tunnel were matched to twelve patients who underwent ACL reconstruction with a hamstring graft and interference screw fixation (IF). The diameters of the bone tunnels were analysed by a multiplanar reconstruction technique (MPR) in a CT scan three months postoperatively. Manual and instrumental laxity (Lachman test, Pivot-shift test, Rolimeter) and functional outcome scores (International Knee Documentation Committee sore, Tegner activity level) were measured after one year follow up.

**Results:**

In the PF group the mean bone tunnel diameter at the level of the joint entrance was not significantly enlarged. One and two centimeter distal to the bone tunnel diameter was reduced by 15 % (p = .001). In the IF group the bone tunnel at the level of the joint entrance was enlarged by 14 % (p = .001). One and two centimeter distal to the joint line the IF group showed a widening of the bone tunnel by 21 % (p < .001) One and two centimeter below the joint line the bone tunnel was smaller in the PF group when compared to the IF group (p < .001). No significant difference for laxity test and functional outcome scores could be shown.

**Conclusion:**

This study demonstrates that press-fit fixation with free autologous bone plugs in the tibial tunnel results in significantly smaller diameter of the tibial tunnel compared to interference screw fixation.

## Background

In anterior cruciate ligament (ACL) reconstruction bone tunnel enlargement is a phenomenon, which has been observed in a variety of graft fixation techniques [1].

Soft tissue grafts lead to a larger increase in bone tunnel diameter in comparison to bone-tendon-bone grafts [2,3]. It has been proposed that graft fixation more closely to the joint line may reduce bone tunnel widening [4]. Adversely, a larger extent of bone tunnel diameter was shown, when using bioabsorbable screws compared to an extracortical fixation at the tibial side [5, 6].

Although bone tunnel enlargement does not appear to influence primary graft stability [7], in case of revision it may make the procedure more difficult to perform, as two-stage revision surgery with bone grafting and delayed ligament reconstruction may be necessary [8–10].

Therefore surgical techniques for ACL reconstruction reducing the phenomenon of bone tunnel widening are required [11].

Press-fit fixation for ACL reconstruction has shown good clinical results with different types of grafts [12–18]. Jagodszinski et al. reported a decrease of the tibial bone tunnel diameter for a tibial press-fit fixation technique using a xenogenic bone cylinder [19].

However reports about bone tunnel enlargement for quadriceps tendon bone pressfit technique using an autologous tibial bone plug are rare [20–22].

Therefore the purpose of this study was to investigate if a tibial press-fit fixation with a free bone block, as a biologic fixation device near to the joint line, can reduce bone tunnel enlargement in fixation of a soft tissue graft.

The hypothesis was that press fit fixation using autologous bone material in the tibial bone tunnel leads to less bone tunnel enlargement than an absorbable interference screw fixation technique.

## Methods

### Study population

From April 2010 until December 2011 twenty-four male patients with primary ACL insufficiency were prospectively enrolled into the study. Twelve male patients underwent a primary ACL reconstruction using a quadriceps tendon autograft in a press-fit fixation (PF). For a comparative matched-pairs analysis twelve male patients, who underwent ACL reconstruction using a standardized technique with quadruple semitendinosus graft with interference screw fixation (PLDLLA; MEGAFIX® C; Karl Storz AG) (IF) were selected as a control group. All patients underwent surgery within three months after injury. Graft choice was primarily a result of each patient’s preference. All procedures were performed by a single surgeon (senior author). Patients were matched according to age (radius of two years for ages 18–20, three years for age 20–30, and five years for patients > 30)), presence of an accompanying meniscus tear or cartilage injury and additional meniscus or cartilage surgery (Table 1). Only male patients were included in the study, female patients were excluded because of the CTs for ethical reasons. Patients with accompanying ligament injuries (collateral ligaments, posterior cruciate ligament) were excluded. The study was approved by the ethical committee of the University Witten / Herdecke, Germany. A written informed consent was obtained from all study patients.

**Table 1:** Comparison of the matched pair groups

Matched pairs	Age (yrs.)	Associated injuries	Associated surgery	Graft thickness
PF1/IF1	18/20	−/−	−/−	8.5/9.0
PF2/IF2	18/18	MT/MT	PMR/PMR	8.0/8.0
PF3/IF3	51/46	MT/MT	−/−	8.0/8.0
PF4/IF4	29/32	−/−	−/−	9.0/8.5
PF5/IF5	39/34	MT/MT	−/−	8.0/7.5
PF6/IF6	22/25	CD,MT/CD,MT	PMR/PMR	9.0/9.0
PF7/IF7	24/21	MT/MT	MR/MR	9.0/8.5
PF8/IF8	26/29	MT/MT	−/−	8.5/8.0
PF9/IF9	22/25	−/−	−/−	8.5/9.0
PF10/IF10	20/22	−/−	−/−	9.0/9.0
PF11/IF11	28/25	MT,CD/MT,CD	−/−	8.5/8.0
PF12/IF12	21/24	MT/MT	−/−	8.0/8.5

MT = meniscal tear

CD = chondral damage

PMR = partial meniscus resection

MR = meniscal repair

### Surgical technique

#### Press-fit fixation technique with a quadriceps tendon graft

In group I (PF) ACL reconstruction was performed using an autologous quadriceps tendon graft with a bone block of 2 cm in length and a free tendon of 5 cm. The diameter of the bone block was 9.4 mm, the diameter of the free tendon end ranged between 8 and 9 mm. A femoral bone tunnel of 9.0 mm diameter was drilled while the tibial bone tunnel was created using a trephine of 9.5 mm outer diameter (Richard Wolf, Knittlingen, Germany). The graft was introduced with the bone block placed into the femoral bone tunnel, while the soft tissue end of the graft was placed on the tibial side. Femoral press-fit fixation was achieved by tapping the bone block into the femoral canal. The sutures at the free tendon end of the graft (#2 Ultrabraid® suture, Smith & Nephew, Inc.) were tied over a bone bridge at the distal end of the tibial tunnel. To assure press fit fixation of the graft in the tibial tunnel the proximal end of the cylindrical bone block harvested from the tibia was tapped next to the graft tissue into the tibial tunnel to about 5-10 mm distal to the tibial articular cortex.

#### Interference screw fixation technique with a quadruple hamstring tendon graft

In group II (IF) a quadrupled hamstring graft was used (grafts between 7.5 and 9 mm were used in the study population). The bone-tunnels were created in a two-incision technique. The tibial tunnel was drilled one centimeter less then the graft size and than dilated accordingly until matching the graft diameter. A polylactide acid interference screw (PLDLLA; MEGAFIX® C; Karl Storz AG) with a length of 28 mm in 5 cases, and 23 mm in 7 cases both with a diameter 9 mm was used for graft fixation on the tibial side. The interference screw was placed 5 to 10 mm distal to the tibial articular cortex. Femoral fixation was achieved using an interference screw an additional endobutton.

### Rehabilitation

All patients followed the same standardized rehabilitation protocol (Table 2).

**Table 2:** Rehabilitation protocol

day 0-5	• Immobilization in a 0° splint
	• Lymphatic drain, cryotherapy
	• Three point gait, partial-weight bearing (up to 15 kg)
	• Active/passive range of motion 0/0/60
day 5 -21	• Active/passive range of motion 0/0/90
	• Three point gait, partial-weight bearing (up to 20–30 kg) in a brace
	• Lymphatic drainage, cryotherapy
	• Quadriceps strengthening
day 21-42	• Full weight-bearing with brace
	• Free active/passive range of motion
	• Quadriceps strengthening
	• Cycling, ergometer
week 6-12	• Muscle-strength training
	• Swimming, aquajogging
week 13-26	• Continuing strengthening exercises
	• Running if tolerated
6-9 months	• Return to sports if muscle strength is above 90° of the contralateral side

### Bone tunnel measurements

Three months after surgery all patients underwent a 16 slice multidetector CT scan of the affected knee (Gemini GXL 16, Philips Medical Systems, Eindhoven, NL). Each CT scan generated 350 primary images. 120 KV and 80 mAs were applied and the thickness of the slices was 1 mm. FOV of 20 cm and reconstruction matrix of 512 x 512 yielded an in plane resolution of 0.4 mm. Reconstruction involved a soft tissue and a high resolution bone algorithm, furthermore a 3D surface rendering and multiplanar reformatting in the coronal and sagittal plane. The DICOM datasets were analyzed in Osirix v.5.6. using multiplanar reformatting perpendicular to the individual axis of the drilled channel.

### Image data analyses

**Fig. 1:**
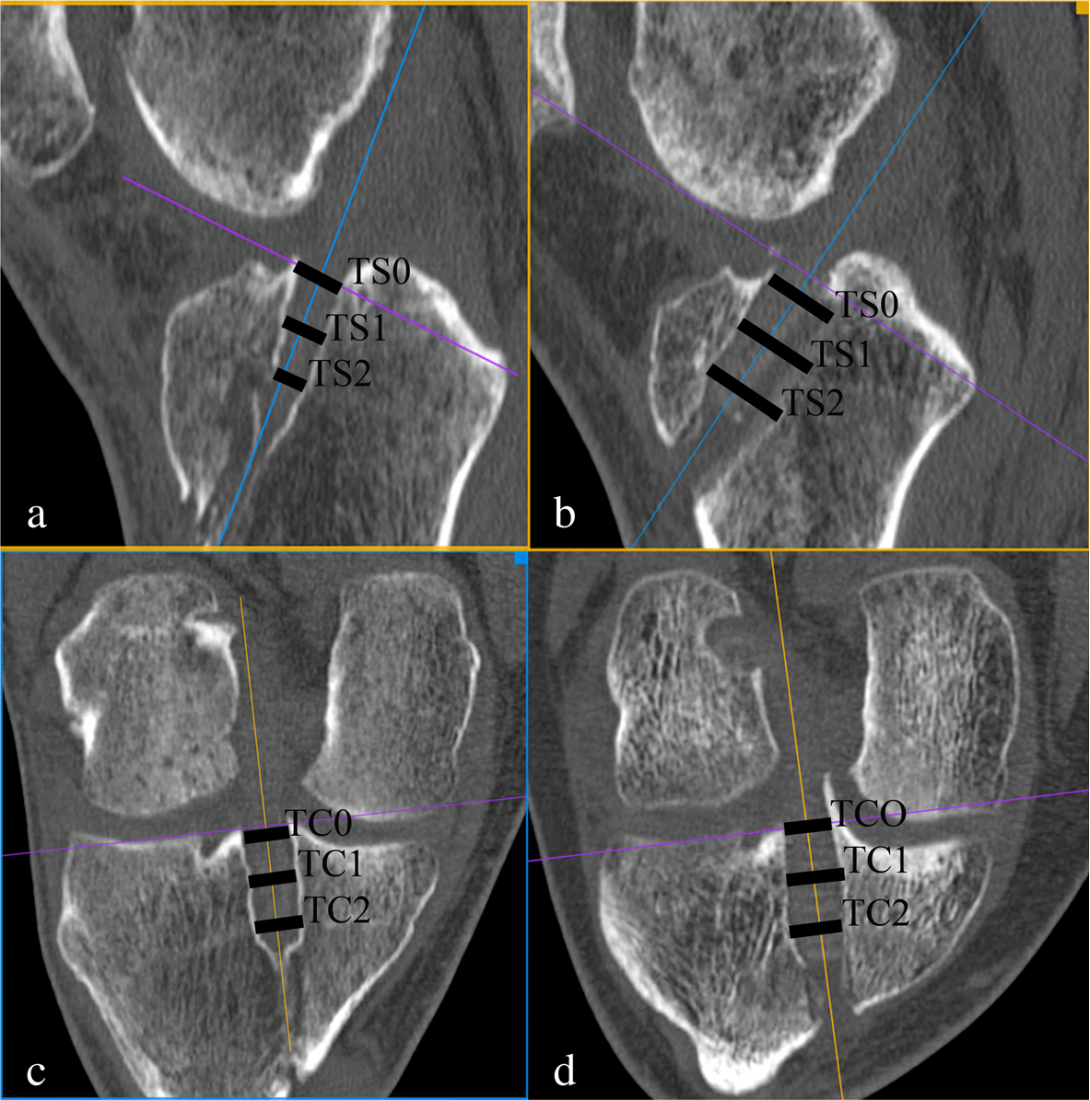
Measurement of the bone tunnel diameter. PF group parasagittal (**a**) and paracoronar (**c**); IF group parasagittal (**b**) and paracoronar (**d**). Three measurements were performed, starting at the level of the tibial cortex (TS0; TC0) as well s 1 cm (TS1; TC1) and 2 cm (TS2; TC2) distal from the cortex.

Parasagittal and paracoronar multiplanar reformations (MPRs) perpendicular to the sagittal and coronal axis of the femoral and tibial bone tunnel were generated. Three measurements were performed, starting at the level of the tibial cortex (TS0; TC0) as well s 1 cm (TS1; TC1) and 2 cm (TS2; TC2) distal from the cortex (Fig. 1). An independent radiologist (JMH, blinded) performed all measurements.

From the measured values at the level of the cortex (TC0; TS0) an arithmetical mean (T0) was formed and the values 1 and 2 cm distally (TC1; TS1 and TC2; TS2) a second arithmetical mean (T1) was summarized.

These bone tunnel diameters T0 and T1 were compared between the two groups (PF and IF), For the PF group the diameters T0 and T1 were compared with the diameters of the trephine used during the surgery, in the IF group it was compared to size of the interference screw.

In both groups, the tunnel morphology and bony integration next to the free autologous bone block or interference screw were analyzed. This was done by visual analysis of the CT layers by an independent radiologist. Criteria for a bony healing were no sclerotic area between the free bone block and bone tunnel and homogeneous representation of the bony structure between the bone block and the tunnel spongious bone. In the IF group the sclerosis zone of the tunnel and the tunnel morphology next to the interference screw were determent.

#### Postoperative evaluation

Knee stability was assessed with, Lachman test (graded negative, 1+, 2+ and 3+), the pivot-shift test (graded negative, (+) glide, + and ++), and an instrumental laxity measurement was performed using the Rolimeter (Aircast Europa GmbH, Neubeuern, Germany),

Further subjective and objective International Knee Documentation Committee score (IKDC) and Tegner activity level were measured at follow up.

### Statistics

Statistical analysis was done using SPSS 21.0 software package (SPSS, Inc. Chicago, Illinois).

Within the matched pair groups bone tunnel diameters were compared to the reamer size used intraoperatively (paired t-test). Furthermore, bone tunnel dilatation was compared between both treatment groups (PF vs. IF; matched pairs; paired t-test). These comparisons were repeated for both T0 and T1. Due to multiple comparison p-value for significance was set at 0.01.

To determine interobserver variability ten CT scans were randomly selected and reviewed by a second observer. The intraclass correlation coefficient (ICC) was performed, using an absolute agreement and a two-way random effect model, in which subjects and raters are considered random effects.

In a post hoc sample size estamination 24 patients achieved a power of 0,8. Alpha level for statistical significance was set at p < .025.

Further Lachman test, pivot-shift test, instrumental laxity measurement, objective IKDC and Tegner (wilcoxon test) and subjective IKDC (unpaird t-test) were compared between the groups.

## Results

Twenty-four patients were enrolled into the study and received a CT scan three month after surgery, twelve in the PF group and twelve in the IF group.

The mean age was 27.4 years (range 18-44years) in the PF group and 25.8 (range 18 – 51 years) in the IF group (p = .08). Ten patients from the PF group and eleven of the IF group were available for the clinical follow up. Mean time from surgery to follow up was 12.7 months (range 6–18 months) in the PF group and 12.8 months (range 11 – 16 months) in the IF group.

**Fig. 2:**
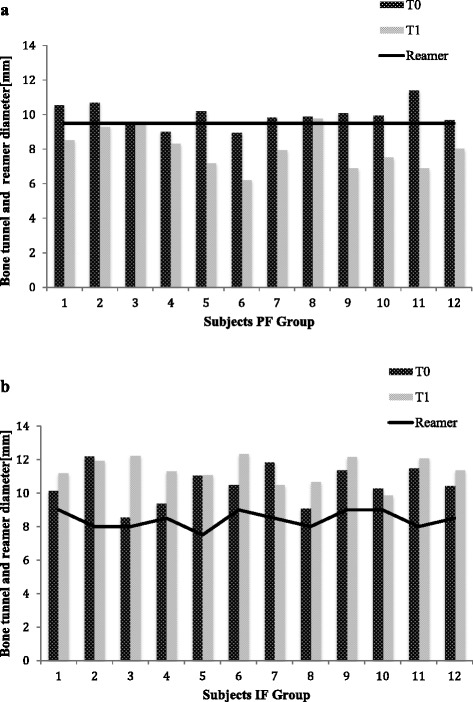
The bone tunnel diameters for each subject of the PF group (**a**) and IF group (**b**) at the level of the cortex (T0) and one and two centimeter distal (T1) three month postoperative shown as columns. The diameter of the intraoperative used reamer is illustrated as a line. Columns below the line indicate a reduction; columns above represent a widening of the tibial bone tunnel diameter.

The results of the CT measurements and the changes in bone tunnel size from surgery to follow up are summarized in Fig. 2.

Within the interval from surgery to follow up, in the PF group the bone tunnel diameter on the articular cortex level (T0) was 5 % larger than the size of the reamer used intraoperatively (MD 9.9 mm ±; SD 0.7 mm; p = .034). One and two centimeter distal to the cortex (T1) the bone tunnel size was 16 % smaller than the reamer used intraoperatively (MD 8.0 mm; SD 1.1 mm; p = .001). In the IF group the bone tunnel was significantly enlarged at the level T0 by 14 % in average (MD 10.5 mm; SD 1.1 mm; p = .001) and at the level T1 by 21 % in average (MD 11.4 mm; SD 0.8 mm; p < .001) (Table 3).

**Table 3:** Mean values and SD of bone tunnel diameter, size of intraoperative reamer and bone tunnel widening

		tunnel diameter [mm]	reamer/screw size [mm]	tunnel widening	p value
PF	T0	9.9 ± 0.7	9.5	+5 %	.034
	T1	8.0 ± 1.1	9.5	−15 %	.001
IF	T0	10.5 ± 0.8	9.0	+14 %	.001
	T1	11.4 ± 1.1	9.0	+21 %	<.001

**Fig. 3:**
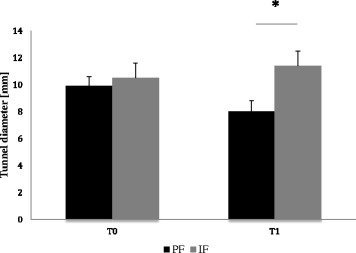
Mean bone tunnel diameters after follow up at the level of the joint line (T0) and one and two centimeter distally (T1) three month after surgery. The IF group showed a higher mean bone tunnel diameter than the PF group at T0 and T1, at T1 it was statistically significant.

Three month after surgery, in the IF group the mean bone tunnel diameter at the level T0 and T1 was larger than in the PF group. At the level T1 this was statistically significant (p < .001) (Fig. 3). The average ICC was 0.84 (range 0.69 – 0.89).

**Fig. 4:**
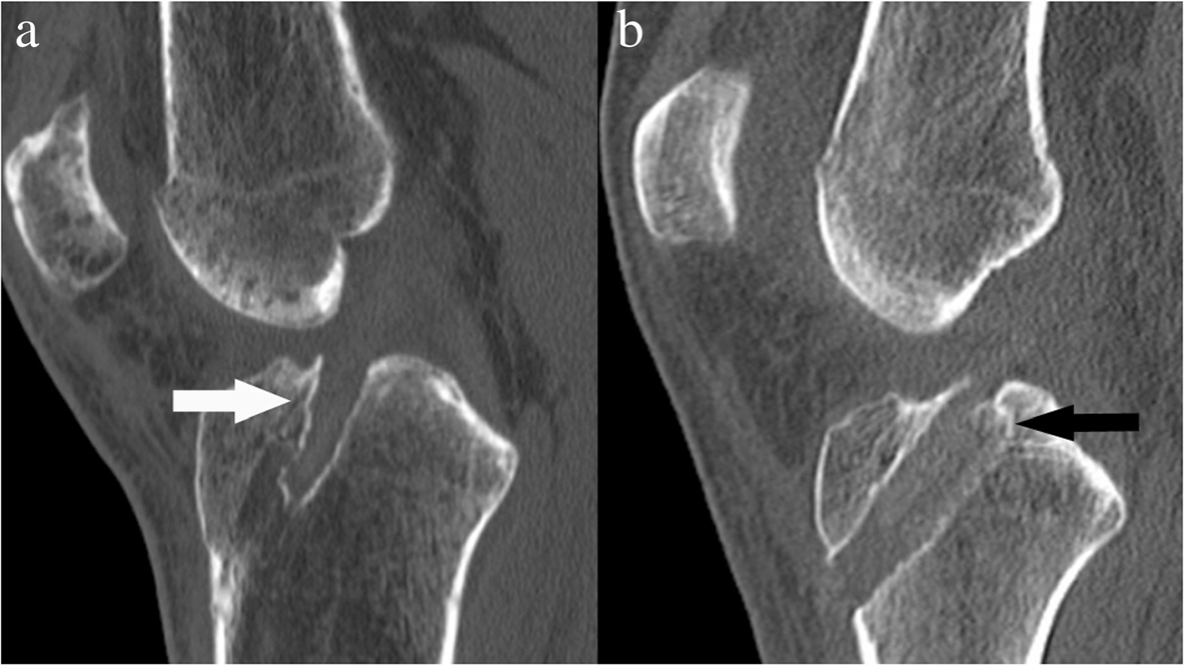
Analysis of bony integration and bone tunnel morphology in the CT scan three month postoperatively. In the PF Group (**a**) all cases of the free autologous bone block (white arrow) showed bony integrated with homogenous spongeous bony structure and no sclerosis between bone block and tunnel wall. In the IF group (**b**) for all cases a tough-shaped bone tunnel dilatation immediately adjacent to the interference screw and a sclerosis zone between the inner bone tunnel and the interference screw was observed (black arrow).

The analysis of the integration of the autologous free bone block of the PF group showed a fully bony integration in each case (Fig. 4a). In the analysis of the bone tunnel in the IF group showed a trough-shaped bone tunnel enlargement in all cases directly adjacent to the interference screw (Fig 4b).

The clinical results are demonstrated in Table 4. There were no statistical significant differences for Lachman test, pivot-shift test, instrumental laxity measurement, subjective and objective IKDC and Tegner activity level between the two groups at the time of follow up.

**Table 4:** Subjective and objective results at follow up

		Mean and SD or n (%)		p value
		PF group (n = 10)	IF group (n = 11)	
Lachman test	negative	9(90)	9(82)	.60
	1+	1(10)	2(18)	
Pivot-shift Test	negative	8(80)	7(64)	.48
	(+)	1(10)	3(27)	
	1+	1(10)	1(9)	
Rolimeter		1.5 (0.9)mm	1.7(1.1)mm	.15
IKDC objective	A	8(80)	7(64)	.32
	B	2(20)	2(18)	
	C		2(18)	
IKDC subjevtive		89.5(8.3)	85.3(9.0)	.8
Tegner activity score		7,4(1.4)	8.2(1.0)	.18

## Discussion

The major finding of our study was that at three months after surgery the diameter of the tibial bone tunnel was significantly smaller in press fit bone wedge group when compared to a group of patients in which an interference screw was used. Therefore we could confirm the hypothesis that press fit fixation with a free autologous bone block, leads to less bone tunnel widening than interference screw fixation.

Bone tunnel widening was reported to increase to almost 50 % of its maximum after six weeks and reaches a steady state after three to six month [3, 23–25]. Therefore in our study it was chosen to perform a CT investigation three month postoperatively.

To our knowledge this is the first study comparing bone tunnel enlargement after primary ACL reconstruction surgery using press-fit fixation technique with an autologous bone block and standard interference screw fixation in the tibial tunnel in a CT based analysis.

At one year after surgery Clatworthy et al. observed a 73 % increased tibial tunnel diameter for hamstring grafts compared to a 2 % decrease for bone-patella tendon bone grafts [3]. Further, L’Insalata et al. reported significantly less dilation of the tibial tunnel for a patella tendon graft compared to a hamstring graft [26]. Among biological and mechanical factors this may be explained by the bony ingrowth of the patellar tendon bone block. Therefore autologues bone blocks in the tibial tunnel seem to reduce bone tunnel enlargement. Also a tibial bioabsorable interference screw, which was expected to reduce bone tunnel enlargement [27, 28] in two studies by Buelow et al. and Dave et. al. showed a greater amount of tunnel expansion for the absorbable screws than for extracortical fixation techniques [5, 6]. As shown in our study, a tibial press-fit fixation close to the joint line with autologues bone blocks in combination to an extracortical fixation may be an alternative to screw fixation close to the joint line.

In this study two frequently used fixation techniques for an ACL graft in the tibial tunnel were analyzed. The results revealed that the autologous bone block was securely integrated into bone and the bone tunnel diameter one and two centimeter distal to the tibial cortex was reduced significantly compared to the diameter of the trephine used intraoperatively.

The bone tunnel diameter on the articular cortex level in the PF group did not change significantly. The reason for this could be, that the free bone block was introduced 5 to 10 mm distal to the tibial articular cortex. The tunnel widening in the IF group in this study was similar to data reported by other authors for hamstring tendons in the tibial tunnel fixed with bioresorbable interference screws and anteromedial portal technique [19].

Our results are in line with Kim et al. who showed significantly less bone tunnel widening for press-fit fixation with autologous bone block compared with interference screw fixation (PLLA, Arthrex, Naples, Florida) with an achilles tendon allograft[20]. In contrast to Kim et al. in our population the diameter of the tibial bone tunnel was reduced three month postoperatively. This may be explained by the fact that Kim et al. used a MRI scan device for measuring the bone tunnel diameter. Marchant et al. showed that the CT is the most reliable imaging modality for evaluation of ACL bone tunnels when compared to MRI and radiographs [29]. Matsumoto et al. and Silva et al. also showed a reduction of the tibial bone tunnel one day, for month and one year after ACL reconstruction surgery using the WasherLoc (Arthrotek, Inc., Warsaw, IN) and bone dowel technique for tibial fixation in a CT scan device in a study design without control groups [21, 22].

The clinical relevance of tunnel enlargement is not fully understood. In most studies, as well as in our investigation, bone tunnel enlargement has no influence on various clinical outcome scores or knee laxity [2, 3, 24, 25]. In contrast Moisala et al. reported a correlation between knee laxity and tibial tunnel widening (p = .02) and Jarvela et al. showed a significant positive correlation between tunnel enlargement and anterior as well as rotational knee laxity [30].

Bone tunnel enlargement plays an important role in revision surgery. In ACL revision surgery one stage and two stage procedures are performed. Advantages of one stage revision are earlier return to normal activity and lower costs. The most important factor in deciding one or two stage revision is the location and the size of the existing bone tunnels. With a bone tunnel diameter of 10-15 mm a two stage procedure is recommended [10, 31, 32]. According to these criteria the tibial tunnel diameter of the PF group in the study population were suitable for a one stage revision procedure.

High risks for graft rupture are reported for young active patients [33, 34]. Data from the Swedish ACL register showed a rate of 4.1 % ACL revision surgery in a five years period, and for 15- to 18-year-old female soccer players 22 % revision or contralateral ACL reconstruction [35]. Especially patients with high risk for subsequent ACL injuries could benefit from ACL reconstruction procedures with techniques minimizing bone loss demonstrated in this study.

In our study all free bone blocks showed bony ingrowth in the bone tunnel without a sclerotic margin between the bone block and the bone tunnel and homogeneous bone morphology between bone block and bone tunnel. The integration into bone of the free bone block in the tibial tunnel seems to be at least comparable to the good results of bony integration observed for the bone block of a bone patella-tendon bone graft in the tibial tunnel [36, 37]. The bony integration of the free autologous bone block and the reduction of the tibial tunnel diameter may be considered as an advantage for revision surgery.

Our observations of bone tunnel widening and embedding of the screw in a trough-shaped recess of the tibial tunnel in the IF group indicates a mechanically contributed tunnel enlargement by drilling the interference screw into the soft bone of the tibial metaphysis. These results confirm the findings by other investigations, who demonstrated that an interference screw not only compresses the graft in the tunnel but also leads to a bone tunnel dilatation by itself [2, 6].

Our study is limited by several factors. First, the use of two different types of grafts, however both grafts quadriceps- and hamstring tendon, demonstrated comparable clinical results in the literature [38–40] In the demonstrated techniques of this study, a soft tissue graft is fixed in the tibial tunnel, the authors were not aware of any data, which showed different tunnel widening for autologous soft tissue grafts. Further the tunnel diameter was only measured at 3 months postoperatively, however further CT scans at other time periods could not be justified for radiation exposure. As no CT scan immediately after surgery was performed, the primary bone tunnel diameter was set equal to the diameter of the trephine used in the PF group and in the IF group to the interference screw. This is a standard method in several previously published studies [2, 3, 24, 26, 41–43]. Iorio et al. showed bone tunnel size measured by CT scan immediately after surgery closely matched the drill bit diameter used intraoperative [44].

An interference screw by itself could produce a widening of the bone tunnel [6], so the primary bone tunnel diameter in the IF group could have been set to low. However, the free bone block in the PF group could have produced a primary bone tunnel enlargement in the same manner as the interference screw.

In addition choice of the graft, which was in most cases decided by patient´s preference, may have an effect on the results. However to create comparable groups each patient of the PF group was matched to an patient of the IF group with same age and same accompanying injuries, all patients were male gender and all procedures were done by one surgeon in the first three month after injury.

Our findings indicate that press-fit fixation may be an alternative for soft tissue graft fixation in the tibial tunnel, especially for patients with a high risk of reinjury. Further studies investigating press-fixation with other soft tissue grafts are needed.

## Conclusion

Press-fit fixation technique using a free autologous bone block and an additional suture fixation with a bone bridge at the distal end of the tibial tunnel, as demonstrated in this study, leads to a reduction of the diameter of the bone tunnel after three months. Our findings confirm that press-fit fixation may result in less bone loss in the tibial bone tunnel and may make revision surgery easier and reduce the necessity of two-stage revision surgery.

## Abbreviations

ACL: Anterior crucriate ligament
FOV: field of view
ICC: Intraclass correlation coefficient
IF: interference screw fixation
IKDC: International Knee Documentation Committee score
MPR: multiplanar reconstruction
PF: press-fit fixation
